# The Morphometry of Soft Tissue Insertions on the Tibial Plateau: Data Acquisition and Statistical Shape Analysis

**DOI:** 10.1371/journal.pone.0096515

**Published:** 2014-05-02

**Authors:** Liying Zheng, Christopher D. Harner, Xudong Zhang

**Affiliations:** 1 Departments of Orthopaedic Surgery, University of Pittsburgh, Pittsburgh, Pennsylvania, United States of America; 2 Department of Mechanical Engineering and Materials Science, University of Pittsburgh, Pittsburgh, Pennsylvania, United States of America; 3 Department of Bioengineering, University of Pittsburgh, Pittsburgh, Pennsylvania, United States of America; University of Pittsburgh, United States of America

## Abstract

This study characterized the soft tissue insertion morphometrics on the tibial plateau and their inter-relationships as well as variabilities. The outlines of the cruciate ligament and meniscal root insertions along with the medial and lateral cartilage on 20 cadaveric tibias (10 left and 10 right knees) were digitized and co-registered with corresponding CT-based 3D bone models. Generalized Procrustes Analysis was employed in conjunction with Principal Components Analysis to first create a geometric consensus based on tibial cartilage and then determine the means and variations of insertion morphometrics including shape, size, location, and inter-relationship measures. Step-wise regression analysis was conducted in search of parsimonious models relating the morphometric measures to the tibial plateau width and depth, and basic anthropometric and gender factors. The analyses resulted in statistical morphometric representations for Procrustes-superimposed cruciate ligament and meniscus insertions, and identified only a few moderate correlations (*R* ^2^: 0.37–0.49). The study provided evidence challenging the isometric scaling based on a single dimension frequently employed in related morphometric studies, and data for evaluating cruciate ligament reconstruction strategies in terms of re-creating the native anatomy and minimizing the risk of iatrogenic injury. It paved the way for future development of computer-aided personalized orthopaedic surgery applications improving the quality of care and patient safety, and biomechanical models with a better population or average representation.

## Introduction

Quantitative morphological studies of musculoskeletal tissue structures generate data and knowledge that can advance basic science as well as clinical applications. Such data and knowledge for the knee joint can aid orthopaedic surgeons in what is referred to as anatomical reconstruction of cruciate ligaments (ACL and PCL). The central tenet of anatomic reconstruction is that a closer replication of the natural anatomy can better restore the knee joint function, and is less likely to cause impingement on or iatrogenic injury to adjacent structures [1]–[5].

Anatomic reconstruction of cruciate ligaments, signified by creating the bone tunnels and placing the substituting grafts where the native ligaments were inserted, presents several intertwined challenges. First, the native ACL and PCL insertions are hard to visualize intra-operatively. Even with the aid of radiographic, arthroscopic, or MRI imaging techniques, the best method for determining tunnel and graft positions during cruciate ligament reconstructions and its reliability have yet to be established [6]–[17]. Second, bone tunnel drilling is a destructive process in a confined space, and is associated with risk of iatrogenic injury to adjacent tissue structures such as cartilage and meniscal roots [18]–[21]. Third, there is considerable inter-person variability in morphology such that a non-specific, “one-size-fits-all” surgical approach would risk of tunnel misplacement and iatrogenic injury [22].

Accurate quantitative knowledge of the morphometrics of joint tissue structures in the knee and their relations as well as variations is the key to engineering innovations to aid surgeons in addressing the above challenges. Previous studies of cruciate ligament and meniscus insertion morphology, however, fell short on generating data or knowledge. Most quantitative findings have been reported in two-dimensional (2D) planes (i.e., the sagittal, coronal or axial plane) [11], [15], [23]–[29]. The morphometric approach used in these studies is typified by statistical analysis of the linear distances, angles, or distance ratios. Analyses of a limited set of linear distances, ratios, or angles frequently fail to capture the complete spatial arrangement of the anatomical landmarks on which the measurements are based [30]. A few observational studies have described the locations of the meniscal root attachments in relation to bony or soft tissue landmarks [31]–[33], but the location measures were not scaled or normalized by any tibial measure to elicit potential invariant or common morphological features. The shape variability of cruciate ligament insertions has been documented largely by qualitative descriptions [28], [29], [34], [35]. The ACL tibial insertion sites were found to be triangular or oval in most specimens in some studies [22], [36], and to be more variable than that of the femoral attachments in others [24], [28], [34]. The tibial PCL insertion site was described as trapezoidal in one study [35], but more various gross appearances were noted in other studies [29], [37]. Meniscal root morphology information was relatively sparse in the literature, with irregular and varied shapes illustrated in a limited number of qualitative anatomy studies [31], [32], [37].

The goal of this study was to characterize the morphometrics of cruciate ligament and meniscal root insertions on the tibial plateau and their inter-relationships. We digitized the outlines of cruciate ligament and meniscal root insertions along with the medial and lateral cartilage on 20 specimens, and co-registered the digitized data with CT-based tibia bone models. We combined Generalized Procrustes Analysis (GPA) and Principal Components Analysis (PCA) to first generate a geometric consensus based on tibial cartilage and then determine the means and variations of insertion site shapes, sizes, locations, and their inter-relationships. We examined the *geometric morphometrics*, which, unlike the conventional morphometrics (e.g., length, width, and area), retain geometrical features throughout the analysis, allowing shapes to be quantitatively expressed in a multivariate manner and morphological differences and variabilities better visualized.

## Methods

### Ethics Statement

We obtained approval from University of Pittsburgh Committee for Oversight of Research and Clinical Training Involving Decedents (CORID) for the use of cadaveric specimens in this study with the need for donor consent waived (Approval No. 305).

### Data Acquisition

**Figure 1:**
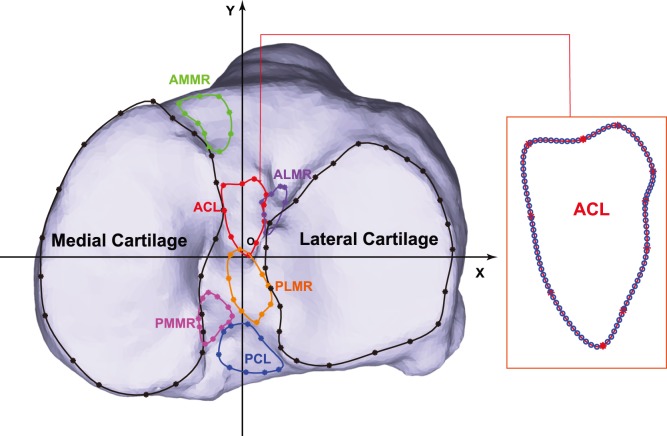
The digitized cartilage and insertion site outlines mapped onto the CT-based 3D tibia model. The digitized points (asterisks) were spline-fitted, generating 100 equidistant points (circles on the close-up view of ACL insertion outline) on the fitted outlines to facilitate the subsequent analyses. The coordinate system shown was defined based on Principal Components Analysis (PCA) of the cartilage outline points.

Twenty cadaveric tibias (10 left and 10 right unpaired knees; 11 from men and 9 from women; mean age at death: 61±5 years) were used in this study. All epithelial, subcutaneous, and muscular tissues were removed from the tibias. High-resolution CT scans of the tibias were taken with slice spacing of 0.625 mm and 3D bone models of the tibias were created in Mimics (Materialise Inc., Belgium). A Polaris Spectra optical tracking system (Northern Digital Inc., Ontario, Canada), with a manufacturer-reported accuracy of ±0.25 mm, was used to digitize the outlines of the medial and lateral tibial cartilage, and the ACL, PCL, anterior-medial, anterior-lateral, posterior-medial and posterior-lateral meniscal root (AMMR, ALMR, PMMR and PLMR) insertion sites. The same experimenter performed all the digitization under the careful supervision of an experienced orthopaedic surgeon. The intra-experimental repeatability was assessed by having the experimenter digitizing the same specimen twice on two different days. The 3D coordinates of anatomical landmarks and pseudo-landmarks (described below) on the cartilage outlines were used to calculate the intraclass correlation coefficients (ICCs), which ranged from 0.94 to 0.99. The digitized outlines were mapped onto the CT-based 3D tibia models with a fiducial registration error smaller than 2% (Fig. 1) [38]. A closed spline was fitted to each outline, resulting in 100 equidistant discrete points to represent the outline (see Fig. 1 for an illustration of a closed spline of an ACL insertion site outline) [39].

### Coordinate System and Landmarks

**Figure 2:**
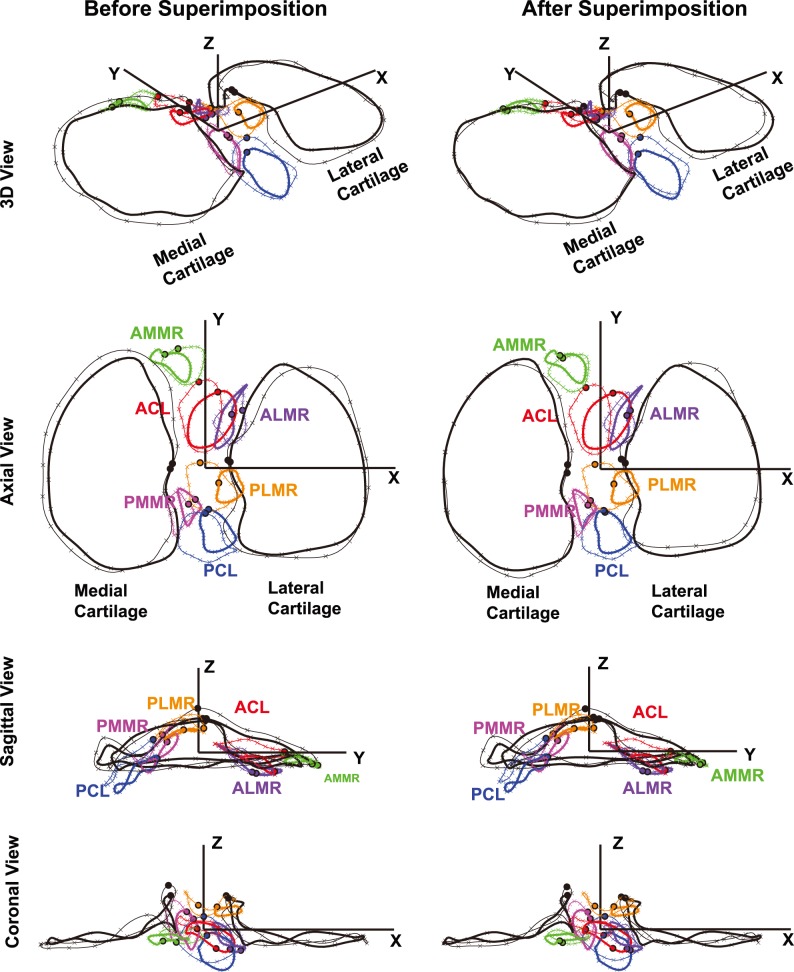
The effect of Procrustes Superimposition illustrated by one pair of tibias. One cartilage configuration served as the base (thick) and another as the target (thin). Six tissue structure insertions in various views before (left column) and after (right column) superimposition are shown as well.

A three-dimensional (3D) coordinate system was established for each tibia model based on its digitized and mapped cartilage outlines. The origin of the coordinate system was first defined to be the midpoint of the medial and lateral cartilage centroids. A Principal Components Analysis (PCA) was then performed on the equidistant discrete points representing the cartilage outlines (200 points in total). The X-axis was the first principal component axis passing the origin and pointing laterally. The Y-axis was orthogonal to the X-axis, passing the origin and pointing anteriorly (Fig. 1 and Fig. 2). To make the Z-axis always point proximally, the coordinate system was a right-handed system for the right tibia and a left-handed system for the left tibia (Fig. 2).

In order to quantitatively describe the geometry of each insertion site contour, two types of landmarks, anatomical landmarks and pseudo-landmarks, were defined in a consistent manner and order so that they corresponded to each other from specimen to specimen. For the medial/lateral tibial cartilage, the medial/lateral tibial eminence apex (the most prominent point on the tibial spine) was identified as the anatomical landmark where the experimenter started and ended the digitization (black solid circles in Fig. 2). Along the fitted cartilage outline (clockwise for the right tibias and counterclockwise for the left tibias), nineteen equidistant points (i.e., every 5^th^ discrete point) were selected as pseudo-landmarks (thin black cross marks in Fig. 2). For the insertion site outlines (ACL, PCL, AMMR, ALMR, PMMR and PLMR), the starting pseudo-landmark was defined as the point at which the line connecting the insertion centroid to the origin of the coordinate system intersected the insertion outline anteriorly in the X–Y plane (black circles with colored filling, Fig. 2); similarly, nineteen additional equidistant pseudo-landmarks were selected along the insertion contours, clockwise for the right tibias and counterclockwise for the left tibias (thin colored cross marks in Fig. 2).

### Generalized Procrustus Analysis (GPA) of Tibial Cartilage Outlines

Cartilage outlines for all 20 tibias were optimally aligned using GPA. GPA is an iterative process of applying Procrustes Superimposition to all possible pairs of configurations–a configuration here refers to a set of cartilage outline landmark coordinates in a pre-defined order. For each cartilage configuration pair, one configuration served as the base and the other as the target. Procrustes Superimposition matches the target configuration onto the base, centering, rotating and uniformly scaling the target configuration to minimize the shape difference (Fig. 2). The shape difference is quantified by the Procrustes Distance (PD) between the base and the superimposed target [40], a dimensionless measure that takes the following form:
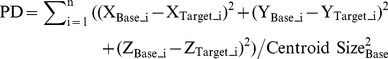
(1)where “n” is the number of landmarks of the configuration (n = 40 for each tibial cartilage configuration); centroid size is a measure of size independent of shape (i.e., centroid size can change without changing shape or vice versa), defined as:



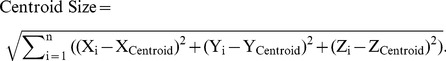
(2)For multiple configurations as in the current study, GPA identified the reference or overall base configuration as one with the smallest overall PD to all others (i.e., the 19 remaining tibial cartilage configurations). The 19 remaining configurations were then Procrustes-superimposed onto this selected reference and their insertion sites transformed accordingly by the same translation, rotation, and scaling rules. The advantage of such a superimposition is that it does not cause any shape distortion. The mean or the most representative tibial cartilage shape could then be created by connecting the average locations of corresponding landmarks (those of the same index) on superimposed cartilage outlines. The reader is referred to [40], [41] for a comprehensive treatment of GPA.

### Shape, Size, Location, and Inter-relationship

The shape variability of cruciate ligament and meniscal root insertion sites was evaluated by individual GPA’s on the already Procrustes-superimposed (based on the cartilage) insertion outlines. The mean shape for each insertion site was created by connecting twenty average locations of the corresponding landmarks.

The 2D areas of the insertion sites were calculated as the projected areas of the outlines on the X–Y plane. The coordinates of the insertion site centroids were expressed in the 3D coordinate system established on each tibia. The distances between the centroids of two adjacent insertion sites were calculated in 3D space as well as in the X–Y plane and the Z direction. The closest distances between two adjacent insertion boundaries were calculated in 3D as well. These distances defined the geometric inter-relationships between the insertion sites.

### Statistical Tests and Regression Models

To examine the effects of GPA on tibal cartilage shape and size, insertion site shape, size, and inter-relationship, paired Student’s t-tests were performed on the following morphometric measures before and after GPA: (1) the PDs of individual tibial cartilage configurations to the “average” configuration; (2) the 2D (in X–Y plane) areas of the tibial medial and lateral cartilage; (3) the 2D insertion site areas; (4) the relative positions between the centroids of two adjacent insertions; and (5) the closest 3D distances between adjacent insertion site boundaries.

To characterize the variability of insertion site centroid location, PCA was performed on the post-GPA centroid coordinates, identifying the major and minor axes of greatest variability in the data while generating 99% confidence ellipses. In addition, PCA was applied to the equidistant landmarks on the outlines of cartilage and insertion sites after superimposition to ascertain the landmark position variability individually and the shape variability collectively.

Lastly, regression analyses were conducted in search of simple ‘parsimonious’ models (with no more than 2 predictors) relating the tibial plateau dimensions, basic anthropometrics and gender factors to the morphometric measures. Particular attention was paid to the morphometric measures that might be important decision variables in cruciate ligament reconstructive surgery such as insertion site areas and centroid locations, and distances between insertion centroids. The intent for this series of analyses was two-fold: (1) to explore how well the morphometrics of soft tissue insertions inside the knee joint are correlated with variables that are practically more measurable (i.e., bony dimensions measured by clinical X-ray, anthropometrics); (2) to assess the uncertainty associated with utilizing these relations in tools or systems for aiding surgeons in identification of the insertion sites [42].

## Results

**Figure 3:**
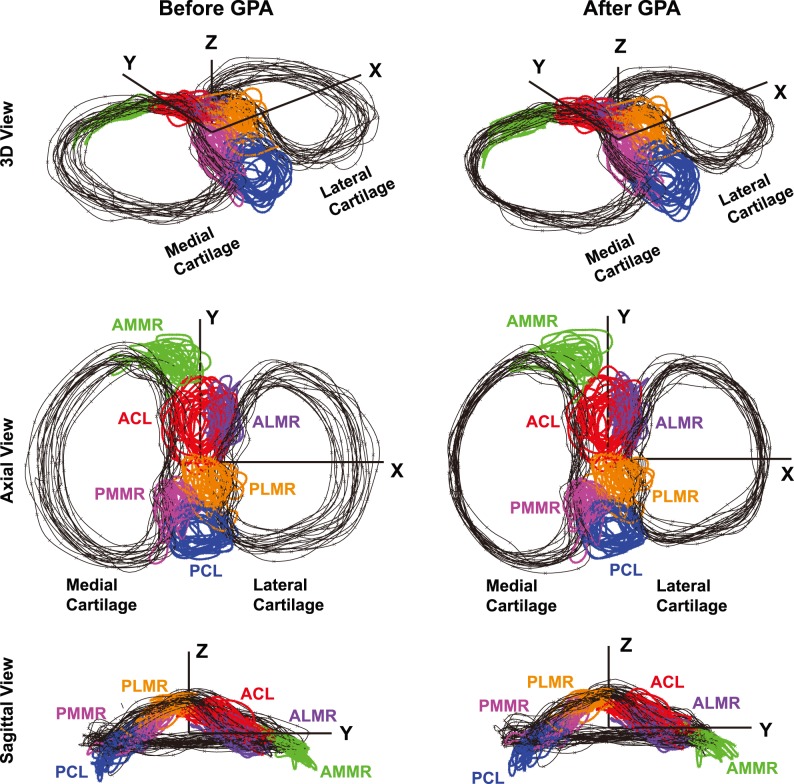
The outlines of tibial cartilage and six insertion sites before and after cartilage-based GPA. The number of tibia samples is 20.

The Generalized Procrustes Analysis (GPA) of the tibial cartilage (Fig. 3) significantly (p<0.001) reduced the Procrustes Distances (PDs) from individual cartilage shapes to the average shape: the mean (± SD) was 0.017 (±0.006) prior to the GPA and 0.010 (±0.005) after. The 2D medial and lateral cartilage areas had minimal changes in the means, whereas their standard deviation (SD) and coefficient of variation (CV) values decreased substantially (Table 1), reflecting a size-uniformity effect of the scaling involved in GPA.

**Table 1:** The 2D (X–Y Plane) areas of tibial cartilage and insertion site outlines.

	Before GPA		After GPA	
	Mean ± SD (mm^2^)	CV (%)	Mean ± SD (mm^2^)	CV (%)
**Medial Cartilage**	1210.8±133.7	11	1221.9±102.8	8
**Lateral Cartilage**	1060.6±166.5	16	1063.7±60.9	6
**PCL**	79.9±18.9	24	80.2±15.8	20
**ACL**	115.0±40.5	35	114.9±32.1	28
**PLMR**	49.6±25.0	50	49.4±22.7	46
**PMMR**	30.8±12.7	41	30.5±11.2	37
**ALMR**	31.2±15.4	49	31.3±14.3	45
**AMMR**	35.8±19.6	55	37.0±22.6	61

The Procrustes superimposition based on the cartilage had a much lower size-uniformity effect on the insertion site areas: the 2D insertion site areas had much greater inherent variations but much smaller variation reductions due to GPA as compared to the cartilage areas (Table 1). The superimposition did not have any marked effect on the distances between insertion site centroids, nor on the closest distances between the boundaries of adjacent insertion sites: the average before-and-after differences were 0.1 mm (see Table 2) and 0.05 mm (see Table 3), respectively.

**Table 2:** Inter-relations between insertion centroids measured by 2D (X–Y Plane) distance, Z position (+ distal; − proximal) and 3D distance (all as Mean ± SD in mm).

	Before GPA			After GPA		
	2D Distance	Z Position	3D Distance	2D Distance	Z Position	3D Distance
**PCL to ACL**	26.6±2.1	5.7±1.6	27.3±2.0	26.8±2.4	5.7±1.6	27.5±2.4
**PCL to PLMR**	11.7±1.6	9.0±1.4	14.8±1.8	11.8±1.7	9.1±1.5	14.9±1.9
**PCL to PMMR**	8.7±0.9	4.4±1.1	9.8±1.1	8.8±0.8	4.4±1.3	9.9±1.1
**ACL to ALMR**	5.7±1.5	−2.6±0.9	6.3±1.5	5.7±1.3	−2.6±0.9	6.3±1.3
**ACL to AMMR**	17.1±3.4	−5.8±2.4	18.2±3.5	17.3±3.4	−6.0±2.3	18.4±3.6

**Table 3:** The 3D closest distances between boundaries of adjacent insertion sites (all as Mean ± SD in mm).

	Before GPA	After GPA
**PCL to PLMR**	4.0±2.6	4.1±2.7
**PCL to PMMR**	1.1±1.0	1.1±0.9
**ACL to ALMR**	0.4±0.5	0.4±0.5
**ACL to AMMR**	6.4±3.7	6.5±3.8

**Figure 4:**
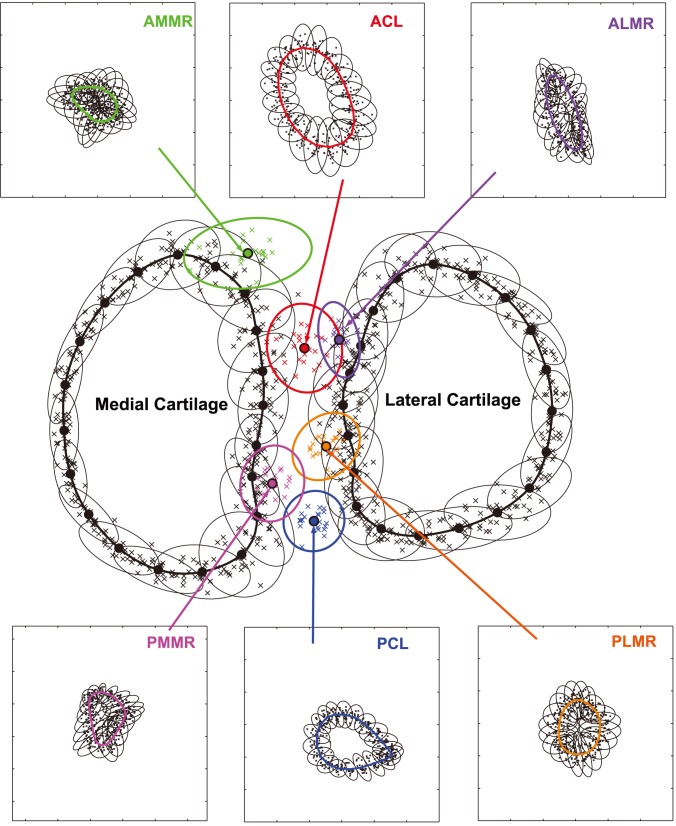
Statistical representations of tibial cartilage, cruciate ligament and meniscus insertion location and shape variability. The thick outlines are the mean or “most representative” shapes (the black thick outlines for tibial cartilage and the colored thick lines in the upper and lower subplots for six insertion sites); the color ellipses are 99% confidence ellipses for the insertion centroid locations resulting from PCA; the thin ellipses centered at the equidistant landmarks (20 on each outline) quantify the landmark position variability individually and the shape variability collectively.

The Principal Components Analysis (PCA) of the insertion centroid location variability on the Procrustes-superimposed tibial plateaus resulted in 99% confidence ellipses quantifying the variability (Fig. 4). The major and minor axis lengths of the ellipses were 13.8 mm and 11.5 mm for ACL, 9.6 mm and 9.1 mm for PCL, 19.8 mm and 10.7 mm for AMMR, 11.8 mm and 6.1 mm for ALMR, 11.8 mm and 9.9 mm for PMMR, and 11.6 mm and 9.1 mm for PLMR. The PCA of equidistant landmarks on the contours of cartilage and insertion sites (on each) generated 99% confidence ellipses centered on the landmarks and thus quantified the variability of landmark locations individually and the variability of contour shape collectively (Fig. 4). Overall, the tibial cartilage contour shape varied more towards the anterior-posterior centerline. The shape variability of the insertion sites, especially of the meniscus roots, was much greater than that of the cartilage, as reflected by the relative sizes between the 99% confidence ellipses and the average shapes.

No strong correlation (*R* ^2^>0.50) was found between the insertion site morphometric measures and the tibial plateau dimensions. The average greatest *R* ^2^ achievable by linear models was 0.21±0.14 with one predictor and 0.30±0.14 with two predictors. A number of parsimonious models with no more than 2 predictors achieving moderate levels of *R* ^2^ (>0.37) are listed as follows:

1. PMMR area = −109+2.83*medial tibial depth (*R* ^2^ = 0.49);
2. ACL area = −157+6.49*lateral tibial depth (*R* ^2^ = 0.37);
3. ACL-to-ALMR centroid distance = −6.64+0.028*weight +0.252*lateral tibial depth (*R* ^2^ = 0.42);
4. PCL-to-PMMR centroid distance = 11.3–0.0566*lateral tibial depth –1.31*gender (*R* ^2^ = 0.39; Male = 0; Female = 1);
5. ACL centroid X-coordinate = 16.9 − 0.0876*weight − 0.267*lateral tibial depth (*R* ^2^ = 0.49);

where the units are mm^2^, mm, and kg for area, distance/coordinate/depth, and weight, respectively.

## Discussion

This study was a systematic effort to quantitatively and statistically characterize the morphometrics–the shape, size, location, and inter-relationship–of soft tissue insertions on the tibial plateau. The primary clinical motivation was anatomical reconstruction of cruciate ligaments (ACL and PCL) with the dual goals of re-creating the native anatomy and minimizing the risk of iatrogenic injury to adjacent tissue structures. The notion of using morphometric information to aid in personalized surgical design and planning is nevertheless general, and so are many aspects of the presented data acquisition and analysis methodology.

Central to our methodology was the Generalized Procrustes Analysis (GPA), which has been employed in fields such as anthropology and zoology [30], [43], [44], but has not previously been applied to morphological studies of musculoskeletal soft tissue structures. In the current study, the GPA played two critical roles. First, the cartilage-based GPA provided a common geometric base across the specimens, effectively serving as a 3D geometric normalization procedure. In conventional morphometric studies, insertion site locations are expressed as percentages of the tibial plateau depth or width or similar representation and the normalization is therefore based on a single dimension [11], [15], [27], [28]. An underlying assumption for single-dimension-based normalization is that the involved morphometric measures adhere to isometric rather than allometric scaling [45]. However, these measures cannot be scaled isometrically–isometric scaling would have resulted in an equitable size-uniformity effect by the GPA between tibial cartilage and insertion sites, which was not observed in the present study (see the CV values in Table 1). Second, the GPA unveiled the true shape variability of the tibial cartilage, not confounded by the size and orientation variability. This quantitative new knowledge of tibial cartilage shape variability may have clinical implications on cartilage repair and tissue engineering, and the design of next-generation tibial components for total knee arthroplasty (TKA).

The average data or representations including average insertion locations, sizes, and shapes, average distances between structures may provide direct, “rule-of-thumb” guidance to orthopaedic surgeons. For instance, surgeons should be made aware of the average clearance–the distance from the ACL or PCL insertion site to the nearest meniscus root (0.4 mm and 1 mm to ALMR and PMMR, respectively; see Table 3). Knowing the average clearance or ‘margin for error’ helps contain the risk of damaging the meniscus roots during an ACL or PCL surgery. Further, our data also suggested that tibial tissue insertion site morphometrics in general and ACL and PCL insertion locations in specific are not highly predictable by tibial dimension measures. There is substantial inter-subject variability–which translates into the uncertainty associated with using average data in a “one-size-fits-all” tunnel placement strategy for reconstructive surgery. The variability information as documented and visualized (as in Fig. 4) in the current study is valuable in that it provides surgeons some sense about the magnitude of uncertainty in managing the potential error or risk during a surgery.

A more advanced and sophisticated way to utilize the morphometric data and knowledge is through computer-aided personalized orthopaedic surgery (CAPOS) applications. Such applications can include pre-operative surgical planning and intra-operative surgical navigation, both based on a patient-specific ‘virtual’ model of the knee. The morphometric representations by the model, depending on the input data, may have different levels of patient-specificity. At one extreme is the scenario with no patient-specific input data, where essentially a population model as illustrated in Fig. 4 is available and would incur the greatest uncertainty. As more patient-specific information is added–from simple dimensions measured by X-ray to a full 3D model reconstructed from MRI, the morphometric uncertainty decreases while the specificity increases. Of note is that even a high-fidelity 3D model obtained from MRI is still associated with uncertainty due to measurement inaccuracy. Algorithms are needed assist in tunnel placement decision-making by calculating the “non-anatomical-ness” defined as the deviation from native insertion and the risk as the probability of damaging adjacent tissue structures.

In addition to aforementioned direct clinical applications, statistical morphometric data also contribute to building better musculoskeletal biomechanical models including multi-body dynamic models and finite element models to address clinical questions. Most prior knee models employed the subject-specific geometry of bone and soft tissues from imaging data (e.g., CT and/or MRI) [46]–[48]. It can however be challenging to obtain accurate shape and location information for the ligament insertion sites and meniscal root attachments from MRI: specific MRI sequences and configurations may be required for different tissue structures [6], [37], [49]. A few modeling studies had to rely on digitization systems to acquire the data *in vitro* [49], [50]. Statistical morphometric data as acquired in the current study can facilitate establishing a rigorous population or “average” representation when measurement means are unavailable or impractical. Furthermore, through computer modeling and simulations, a sensitivity study incorporating statistical data with mean and variability information can be implemented to investigate how morphometric variations affect the mechanical behavior or responses (e.g., tibiofemoral contact pressure).

We recognize that the tibia sample in our study was limited in number and range of variation–the size variation as reflected by the scaling factors in the Procrustes analysis ranged from 0.89 to 1.16 (mean value: 1.01±0.07). While the primary purpose of this study was to establish and demonstrate the methodology, a more robust and diverse sample could potentially strengthen the statistical descriptions of and correlations between morphometric measures, and allow exploration of additional effects such as gender and age.

## References

[1] Plaweski S, Petek D, Saragaglia D (2011) Morphometric analysis and functional correlation of tibial and femoral footprints in anatomical and single bundle reconstructions of the anterior cruciate ligament of the knee. Orthop Traumatol Surg Res 97: S75–79. 21903501 10.1016/j.otsr.2011.07.004

[2] Zantop T, Diermann N, Schumacher T, Schanz S, Fu FH, et al (2008) Anatomical and nonanatomical double-bundle anterior cruciate ligament reconstruction: importance of femoral tunnel location on knee kinematics. Am J Sports Med 36: 678–685. 18296542 10.1177/0363546508314414

[3] Simmons R, Howell SM, Hull ML (2003) Effect of the angle of the femoral and tibial tunnels in the coronal plane and incremental excision of the posterior cruciate ligament on tension of an anterior cruciate ligament graft: an in vitro study. J Bone Joint Surg Am 85-A: 1018–1029. 12783997 10.2106/00004623-200306000-00006

[4] Iriuchishima T, Tajima G, Ingham SJ, Shirakura K, Fu FH (2012) PCL to graft impingement pressure after anatomical or non-anatomical single-bundle ACL reconstruction. Knee Surg Sports Traumatol Arthrosc 20: 964–969. 21935616 10.1007/s00167-011-1680-0

[5] Galloway MT, Grood ES, Mehalik JN, Levy M, Saddler SC, et al (1996) Posterior cruciate ligament reconstruction. An in vitro study of femoral and tibial graft placement. Am J Sports Med 24: 437–445. 8827301 10.1177/036354659602400406

[6] Araujo P, van Eck CF, Torabi M, Fu FH (2012) How to optimize the use of MRI in anatomic ACL reconstruction. Knee Surg Sports Traumatol Arthrosc 21: 1495–1501. 22893266 10.1007/s00167-012-2153-9 PMC3685708

[7] Khalfayan EE, Sharkey PF, Alexander AH, Bruckner JD, Bynum EB (1996) The relationship between tunnel placement and clinical results after anterior cruciate ligament reconstruction. Am J Sports Med 24: 335–341. 8734885 10.1177/036354659602400315

[8] Sullivan JP, Matava MJ, Flanigan DC, Gao Y, Britton CL, et al (2012) Reliability of Tunnel Measurements and the Quadrant Method Using Fluoroscopic Radiographs After Anterior Cruciate Ligament Reconstruction. Am J Sports Med 40: 2236–2241. 22962289 10.1177/0363546512458086

[9] Bernard M, Hertel P, Hornung H, Cierpinski T (1997) Femoral insertion of the ACL. Radiographic quadrant method. Am J Knee Surg 10: 14–21 discussion 21–12. 9051173

[10] Klos TV, Banks SA, Habets RJ, Cook FF (2000) Sagittal plane imaging parameters for computer-assisted fluoroscopic anterior cruciate ligament reconstruction. Comput Aided Surg 5: 28–34. 10767093 10.1002/(SICI)1097-0150(2000)5:1<28::AID-IGS4>3.0.CO;2-P

[11] Amis AA, Jakob RP (1998) Anterior cruciate ligament graft positioning, tensioning and twisting. Knee Surg Sports Traumatol Arthrosc 6 Suppl 1 S2–12. 9608456 10.1007/s001670050215

[12] Pinczewski LA, Salmon LJ, Jackson WF, von Bormann RB, Haslam PG, et al (2008) Radiological landmarks for placement of the tunnels in single-bundle reconstruction of the anterior cruciate ligament. J Bone Joint Surg Br 90: 172–179. 18256083 10.1302/0301-620X.90B2.20104

[13] Kantaras AT, Johnson DL (2002) The medial meniscal root as a landmark for tibial tunnel position in posterior cruciate ligament reconstruction. Arthroscopy 18: 99–101. 11774150 10.1053/jars.2002.25345

[14] Harner CD, Hoher J (1998) Evaluation and treatment of posterior cruciate ligament injuries. Am J Sports Med 26: 471–482. 9617416 10.1177/03635465980260032301

[15] Lorenz S, Elser F, Brucker PU, Obst T, Imhoff AB (2009) Radiological evaluation of the anterolateral and posteromedial bundle insertion sites of the posterior cruciate ligament. Knee Surg Sports Traumatol Arthrosc 17: 683–690. 19308356 10.1007/s00167-009-0770-8

[16] Moorman CT 3rd, Murphy Zane MS, Bansai S, Cina SJ, Wickiewicz TL, et al (2008) Tibial insertion of the posterior cruciate ligament: a sagittal plane analysis using gross, histologic, and radiographic methods. Arthroscopy 24: 269–275. 18308177 10.1016/j.arthro.2007.08.032

[17] Dennis MG, Fox JA, Alford JW, Hayden JK, Bach BR Jr (2004) Posterior cruciate ligament reconstruction: current trends. J Knee Surg 17: 133–139. 15366267 10.1055/s-0030-1248211

[18] Kennedy NI, Michalski MP, Engebretsen L, LaPrade RF (2014) Iatrogenic Meniscus Posterior Root Injury Following Reconstruction of the Posterior Cruciate LigamentA Report of Three Cases. The Journal of Bone & Joint Surgery Case Connector 4: 1–6. 10.2106/JBJS.CC.M.00175 29252579

[19] Abdelkafy A (2012) Protection of the medial femoral condyle articular cartilage during drilling of the femoral tunnel through the accessory medial portal in anatomic anterior cruciate ligament reconstruction. Arthrosc Tech 1: e149–154. 23766987 10.1016/j.eats.2012.05.008 PMC3678619

[20] Lubowitz JH (2009) Anteromedial portal technique for the anterior cruciate ligament femoral socket: pitfalls and solutions. Arthroscopy 25: 95–101. 19111224 10.1016/j.arthro.2008.10.012

[21] Gadikota HR, Sim JA, Hosseini A, Gill TJ, Li G (2012) The relationship between femoral tunnels created by the transtibial, anteromedial portal, and outside-in techniques and the anterior cruciate ligament footprint. Am J Sports Med 40: 882–888. 22302206 10.1177/0363546511434276 PMC3740359

[22] Ferretti M, Doca D, Ingham SM, Cohen M, Fu FH (2012) Bony and soft tissue landmarks of the ACL tibial insertion site: an anatomical study. Knee Surg Sports Traumatol Arthrosc 20: 62–68. 21710110 10.1007/s00167-011-1592-z

[23] Harner CD, Baek GH, Vogrin TM, Carlin GJ, Kashiwaguchi S, et al (1999) Quantitative analysis of human cruciate ligament insertions. Arthroscopy 15: 741–749. 10524822 10.1016/s0749-8063(99)70006-x

[24] Colombet P, Robinson J, Christel P, Franceschi JP, Djian P, et al (2006) Morphology of anterior cruciate ligament attachments for anatomic reconstruction: a cadaveric dissection and radiographic study. Arthroscopy 22: 984–992. 16952729 10.1016/j.arthro.2006.04.102

[25] Siebold R, Ellert T, Metz S, Metz J (2008) Tibial insertions of the anteromedial and posterolateral bundles of the anterior cruciate ligament: morphometry, arthroscopic landmarks, and orientation model for bone tunnel placement. Arthroscopy 24: 154–161. 18237698 10.1016/j.arthro.2007.08.006

[26] Edwards A, Bull AM, Amis AA (2007) The attachments of the fiber bundles of the posterior cruciate ligament: an anatomic study. Arthroscopy 23: 284–290. 17349472 10.1016/j.arthro.2006.11.005

[27] Osti M, Tschann P, Kunzel KH, Benedetto KP (2012) Anatomic characteristics and radiographic references of the anterolateral and posteromedial bundles of the posterior cruciate ligament. Am J Sports Med 40: 1558–1563. 22539538 10.1177/0363546512445166

[28] Edwards A, Bull AM, Amis AA (2007) The attachments of the anteromedial and posterolateral fibre bundles of the anterior cruciate ligament: Part 1: tibial attachment. Knee Surg Sports Traumatol Arthrosc 15: 1414–1421. 17934717 10.1007/s00167-007-0417-6

[29] Tajima G, Nozaki M, Iriuchishima T, Ingham SJ, Shen W, et al (2009) Morphology of the tibial insertion of the posterior cruciate ligament. J Bone Joint Surg Am 91: 859–866. 19339570 10.2106/JBJS.H.00991

[30] Slice DE (2007) Geometric Morphometrics. Annual Review of Anthropology 36: 261–281.

[31] Kohn D, Moreno B (1995) Meniscus insertion anatomy as a basis for meniscus replacement: a morphological cadaveric study. Arthroscopy 11: 96–103. 7727019 10.1016/0749-8063(95)90095-0

[32] Johnson DL, Swenson TM, Livesay GA, Aizawa H, Fu FH, et al (1995) Insertion-site anatomy of the human menisci: gross, arthroscopic, and topographical anatomy as a basis for meniscal transplantation. Arthroscopy 11: 386–394. 7575868 10.1016/0749-8063(95)90188-4

[33] Johannsen AM, Civitarese DM, Padalecki JR, Goldsmith MT, Wijdicks CA, et al (2012) Qualitative and quantitative anatomic analysis of the posterior root attachments of the medial and lateral menisci. Am J Sports Med 40: 2342–2347. 22962297 10.1177/0363546512457642

[34] Tallay A, Lim MH, Bartlett J (2008) Anatomical study of the human anterior cruciate ligament stump’s tibial insertion footprint. Knee Surg Sports Traumatol Arthrosc 16: 741–746. 18481044 10.1007/s00167-008-0552-8

[35] Sheps DM, Otto D, Fernhout M (2005) The anatomic characteristics of the tibial insertion of the posterior cruciate ligament. Arthroscopy 21: 820–825. 16012495 10.1016/j.arthro.2005.04.105

[36] Petersen W, Zantop T (2007) Anatomy of the anterior cruciate ligament with regard to its two bundles. Clin Orthop Relat Res 454: 35–47. 17075382 10.1097/BLO.0b013e31802b4a59

[37] Brody JM, Hulstyn MJ, Fleming BC, Tung GA (2007) The meniscal roots: gross anatomic correlation with 3-T MRI findings. AJR Am J Roentgenol 188: W446–450. 17449741 10.2214/AJR.06.0509

[38] Li K, O’Farrell M, Martin D, Kopf S, Harner C, et al (2009) Mapping ligament insertion sites onto bone surfaces in knee by co-registration of CT and digitization data. J Biomech 42: 2624–2626. 19712934 10.1016/j.jbiomech.2009.06.042

[39] D’Errico J (2012) interparc (http://www.mathworks.com/matlabcentral/fileexchange/34874-interparc).MATLAB Central File Exchange.

[40] Dryden IL, Mardia KV (1998) Statistical Shape Analysis. West Sussex, England: John Wiley & Sons Ltd. 376 p.

[41] Mitteroecker P, Gunz P (2009) Advances in Geometric Morphometrics. Evolutionary Biology 36: 235–247.

[42] Zhang X, Moloney G, Araujo P, Langdale E, Churilla A, et al (2012) Efficacy of an Intra-Operative Imaging Software System for Anatomic Anterior Cruciate Ligament Reconstruction Surgery. Journal of Healthcare Engineering 3: 443–454.

[43] Bookstein F, Schafer K, Prossinger H, Seidler H, Fieder M, et al (1999) Comparing frontal cranial profiles in archaic and modern homo by morphometric analysis. Anat Rec 257: 217–224. 10620751 10.1002/(SICI)1097-0185(19991215)257:6<217::AID-AR7>3.0.CO;2-W

[44] Lawing AM, Polly PD (2010) Geometric morphometrics: recent applications to the study of evolution and development. Journal of Zoology 280: 1–7.

[45] Lleonart J, Salat J, Torres GJ (2000) Removing allometric effects of body size in morphological analysis. J Theor Biol 205: 85–93. 10860702 10.1006/jtbi.2000.2043

[46] Sandholm A, Schwartz C, Pronost N, Zee M, Voigt M, et al (2011) Evaluation of a geometry-based knee joint compared to a planar knee joint. The Visual Computer 1: 161–171.

[47] Guess TM, Thiagarajan G, Kia M, Mishra M (2010) A subject specific multibody model of the knee with menisci. Med Eng Phys 32: 505–515. 20359933 10.1016/j.medengphy.2010.02.020

[48] Pena E, Calvo B, Martinez MA, Doblare M (2006) A three-dimensional finite element analysis of the combined behavior of ligaments and menisci in the healthy human knee joint. J Biomech 39: 1686–1701. 15993414 10.1016/j.jbiomech.2005.04.030

[49] Li G, Gil J, Kanamori A, Woo SL (1999) A validated three-dimensional computational model of a human knee joint. J Biomech Eng 121: 657–662. 10633268 10.1115/1.2800871

[50] Donahue TL, Hull ML, Rashid MM, Jacobs CR (2002) A finite element model of the human knee joint for the study of tibio-femoral contact. J Biomech Eng 124: 273–280. 12071261 10.1115/1.1470171

